# Editorial: Epigenetic drugs and therapeutic resistance for epithelial malignancies

**DOI:** 10.3389/fphar.2023.1208518

**Published:** 2023-05-09

**Authors:** Fangfang Tao, Zhiqian Zhang

**Affiliations:** ^1^ Department of Immunology and Microbiology, Basic Medical College, Zhejiang Chinese Medical University, Hangzhou, Zhejiang, China; ^2^ Department of Human Cell Biology and Genetics, School of Medicine, Southern University of Science and Technology, Shenzhen, China; ^3^ Academy for Advanced Interdisciplinary Studies, Southern University of Science and Technology, Shenzhen, China

**Keywords:** epigenetic modifications, cancer therapy, epigenetic drugs, therapeutic resistance, epithelial malignancies

Epigenetic modifications are widely recognized for their crucial role in the development and progression of cancer, particularly in epithelial malignancies. These changes involve modifications to DNA molecules and their associated proteins that can influence gene expression without altering the DNA sequence itself. Given their heritable and reversible nature, epigenetic modifications have become an attractive target for cancer therapy. In recent years, there has been a growing interest in developing epigenetic drugs that can target specific modifications and potentially overcome therapeutic resistance. Many cancers, such as breast cancer, lung cancer, and colorectal cancer, are some of the most commonly diagnosed epithelial malignancies worldwide. Although significant progress has been made in developing targeted therapies, drug resistance remains a significant challenge, which often results in treatment failure and disease progression. Epigenetic modifications, such as nuclear dynamic, DNA methylation, covalent histone modification, histone variants, and non-coding RNA (ncRNA)—including microRNA (miRNA/miR) and long ncRNA (lncRNA)—have all been shown to play a critical role in the development of therapeutic resistance in cancer.

Epigenetic modifications play a significant role in the development of drug resistance in cancer patients. However, drugs that target these modifications, such as DNA methyltransferase inhibitors and histone deacetylase inhibitors, have the potential to reverse them and restore sensitivity to standard therapies (Steele et al., 2009; Vijayaraghavalu and Labhasetwar, 2018; Bao et al., 2020). One example of a successful treatment is the use of 5-Aza-2′-deoxycytidine (5-aza-D) to reverse cisplatin resistance in bladder cancer cells. This effect is attributed to the demethylation of the HOXA9 gene promoter (Xylinas et al., 2016). The use of such drugs is a promising approach to combat drug resistance, particularly in patients with hematological cancer types.

The focus of this Research Topic is on epigenetic events in epithelial malignancies, particularly their development, properties, and mechanistic studies.

First, Epigenetic alterations are known to play a significant role in the development of epithelial malignancies and can also serve as biomarkers to predict their outcome. In this Research Topic, Ye et al. explore the roles of 84 methylation-related genes (MRGs) modification patterns in prostate cancer (PCa) and tumor microenvironment (TME) diversity, clinicopathological characteristics, and various prognostic regulatory mechanisms. The clinical significance of MRGs is highlighted, offering a new perspective for PCa research and advancing our understanding of TME and immunotherapy. Similarly, Cao et al. investigate the role of gene methylation in Pancreatic adenocarcinoma (PAAD), screening potential anti-cancer small molecule drugs and constructing a prediction model to assess PAAD prognosis. The classification model based on differentially methylated and expressed genes (DMEGs) accurately distinguished between normal and tumor samples, underscoring the potential of epigenetic biomarkers and precision medicine in managing PAAD.

Second, therapeutic resistance poses a significant challenge in cancer treatment, and epigenetic changes have been identified as one of the underlying factors. DNA methylation, histone modifications, and chromatin remodeling are examples of epigenetic alterations that contribute to the resistance of cancer cells to chemotherapy therapy. These modifications can disrupt the expression of genes involved in cell cycle regulation, DNA repair, and apoptosis, ultimately leading to the survival and proliferation of cancer cells. Across these publications, Zhang et al. investigate molecular subtypes of thyroid cancer based on immune cell infiltration, underscoring the role of epigenetic modifications in tumor progression and their potential for immunotherapy. This highlights the significance of considering epigenetic modifications in the development of effective cancer therapies.

The role of lncRNA in epigenetics and therapeutic resistance has garnered increasing attention. LncRNAs regulate gene expression through various mechanisms, including transcriptional and post-transcriptional regulation, chromatin remodeling, and competitive binding with miRNAs. The mechanisms involved in lncRNA-mediated gene expression regulation are complex and diverse. Moreover, lncRNA has been linked to the development of therapeutic resistance. In-depth research on lncRNA is expected to yield new strategies and directions for tumor treatment and prevention. Throughout research, Zhang et al. identified RNF157-AS1 as a key lncRNA associated with both doxorubicin resistance and hepatocellular carcinoma (HCC) prognosis. Furthermore, they developed a four-gene risk model that shows potential for predicting HCC prognosis.

Third, in recent years, high-throughput sequencing technology, public databases, and single-cell sequencing technology have become important tools in studying epigenetics. High-throughput sequencing can quickly and accurately detect changes at multiple levels, such as genome, transcriptome, and epigenome levels, providing valuable insights into epigenetic mechanisms. Public databases, such as TCGA and ICGC, include large-scale datasets of tumor samples, enabling researchers to obtain comprehensive epigenetic information and gain important insights into cancer therapy. Within this Research Topic, multiple studies have utilized data from hepatocellular carcinoma (Cheng et al.; Wang et al.), prostate cancer (Deng et al.), pancreatic cancer (Cao et al.; Cao et al.; Ji et al., 2023), lung adenocarcinoma (Liu et al.; Zhou and Zhao), glioblastoma (Lu et al.), colon adenocarcinoma a (Feng et al.), clear cell renal cell carcinoma (Deng et al.), and associated drug resistance data from GEO, TCGA, ICGC, and GTEx. These databases hold a wealth of valuable information that is yet to be fully explored.

Single-cell sequencing technology is a newly emerging tool that enables high-throughput sequencing of the genome, transcriptome, epigenome, and other information of individual cells with high resolution and accuracy. It allows researchers to study the epigenetic differences between cells, better understand the mechanisms underlying tumor occurrence and development, and develop more precise cancer therapy strategies. In this Research Topic, Deng et al. utilized the single-cell dataset of clear cell renal cell carcinoma to identify 24 cell clusters and marker genes for two different cell types in each cluster. Correlation analysis from the research of Lu et al. revealed that paclitaxel treatment affects neurons and may improve glucose metabolism and modulate immune function in glioblastoma. This study highlights the potential of single-cell sequencing to investigate the pharmacological targets and signaling pathways of paclitaxel in glioblastoma and gain insights into its mechanism of action.

In addition, promising approaches have emerged for overcoming therapeutic resistance in epithelial malignancies, including small molecule drugs, traditional Chinese medicine, and network pharmacology. Small molecule drugs, in particular, have demonstrated great potential in targeting epigenetic modifications, specifically histone acetylation and DNA methylation. By selectively inhibiting the enzymes responsible for these modifications, small molecule drugs can disrupt oncogenic signaling pathways and induce apoptosis in cancer cells. Additionally, combination therapy with small molecule drugs and conventional chemotherapy can enhance treatment efficacy and reduce the likelihood of therapeutic resistance. Traditional Chinese medicine has demonstrated promising results in the treatment of epithelial malignancies. These medicines have multi-target effects that can modulate epigenetic modifications, including histone deacetylation and DNA methylation, inhibiting cancer cell growth and proliferation. Moreover, traditional Chinese medicine injections can improve immune function, enhancing the body’s ability to fight cancer. Network pharmacology, a novel approach that combines computational methods with experimental validation, has also shown promise in identifying the molecular mechanisms underlying drug action. It can help to identify novel targets for cancer therapy and provide insights into the mechanisms of therapeutic resistance. In this Research Topic, Cao et al. describe a study on paeoniflorin (PF), an herbal active ingredient with anti-tumor effects. They identified PF targets and performed gene enrichment analysis to determine the biological processes impacted by PF. This study also identified the most relevant genes to PF treatment and validated the identified targets using PANC-1 and Capan-2 cells. The study found that PF may regulate inflammatory factors through the p38 MAPK signal pathway and may have potential as a natural anti-tumor compound for pancreatic cancer. Similarly, Wang et al. identified active ingredients and related genes of traditional Chinese medicine injections for treating hepatocellular carcinoma (HCC), characterizing two HCC subtypes and identifying important genes such as *SPP1* as an oncogene in HCC. The study suggests that traditional Chinese medicine injections can serve as an important adjuvant treatment modality for HCC.

As research on the role of epigenetics in cancer continues to advance, the potential for epigenetic drugs to overcome therapeutic resistance and improve patient outcomes becomes increasingly clear. Future studies are likely to focus on the development of more specific and effective epigenetic therapies that can target specific modifications and combat drug resistance. Additionally, the integration of high-throughput sequencing technology, single-cell sequencing, and network pharmacology will continue to provide new insights into the underlying mechanisms of tumor development and therapeutic resistance. As personalized medicine and precision oncology continue to advance, the identification of epigenetic biomarkers for predicting patient response to therapy will become increasingly important.
